# *TRAF3IP3*::*FGFR1*: a novel *FGFR1* fusion identified in an aggressive case of acute myeloid leukemia

**DOI:** 10.1007/s00277-025-06494-9

**Published:** 2025-07-01

**Authors:** Xue Chen, Lili Yuan, Xiaoli Ma, Fang Wang, Yang Zhang, Panxiang Cao, Ping Wu, Tong Wang, Jiaqi Chen, Xiaosu Zhou, Hongxing Liu

**Affiliations:** 1 Division of Laboratory Medicine, Hebei Yanda Lu Daopei Hospital, Langfang, China; 2 Precision Medicine Center, Beijing Lu Daopei Institute of Hematology, Beijing, China

## Abstract

Myeloid/lymphoid neoplasms with tyrosine kinase gene fusions (MLN-TK) are rare hematologic malignancies characterized by recurrent kinase rearrangements, including *FGFR1*, often associated with aggressive clinical behavior. We report the first case of acute myeloid leukemia (AML) harboring a novel *TRAF3IP3*::*FGFR1* fusion, identified by whole transcriptome sequencing. The patient, a 35-year-old man, presented with monocytic AML and succumbed to disease within 40 days despite induction chemotherapy. Cytogenetic and molecular profiling revealed a complex monosomal karyotype and a pathogenic *TP53* mutation, both known adverse prognostic markers. The in-frame fusion retained the coiled-coil domain of TRAF3IP3 and the full tyrosine kinase domain of FGFR1, suggesting preserved dimerization and oncogenic signaling. This case broadens the spectrum of *FGFR1*-rearranged neoplasms and highlights the importance of early genomic profiling in aggressive leukemia. It also underscores the potential therapeutic opportunities with *FGFR1*-targeted agents such as pemigatinib.

Myeloid/lymphoid neoplasms with eosinophilia and tyrosine kinase gene fusions (MLN-TK) constitute a rare but clinically significant category of hematologic malignancies. They are defined by recurrent rearrangements involving tyrosine kinase genes—most notably *FGFR1*,* PDGFRA*, *PDGFRB*, *JAK2*, and *FLT3*—that generate constitutively active fusion proteins and promote leukemogenesis [1, 2]. Among these, *FGFR1*-rearranged cases are particularly aggressive and frequently refractory to conventional chemotherapy [3–5]. Here, we report a unique case of *de novo* acute myeloid leukemia (AML) harboring a novel *TRAF3IP3*::*FGFR1* fusion.

A 35-year-old previously healthy male presented with gingival bleeding and fever. Laboratory tests showed anemia (Hb 102 g/L), thrombocytopenia (platelets 15 × 10⁹/L), and a normal white blood cell count (WBC 8.7 × 10⁹/L). Bone marrow aspirate demonstrated hypercellularity with 4.5% myeloblasts, 10.5% monoblasts, and 8% promonocytes, without peripheral or marrow eosinophilia (Fig. 1A). Flow cytometric immunophenotyping identified 63.7% immature monocytic cells expressing CD33, CD96, CD11b, CD11c, CD371, CD123, CD4, partial CD34, CD117, CD13, CD64, CD2, and CD9. The following markers were assessed but found to be negative: HLA-DR, CD7, CD56, CD19, MPO, CD22, cytoplasmic CD3, CD14, CD38, CD15, CD24, CD42b, CD42a, CD36, and CD300e. The lower percentage observed by morphological evaluation may be due to sample dilution during bone marrow aspiration, which can lead to reduced representation of immature cells on the smear. Furthermore, immature monocytic cells may display subtle cytologic features that are difficult to distinguish microscopically, whereas flow cytometry allows for more precise identification based on lineage-specific surface markers. Conventional G-banding analysis was performed on 19 metaphases, of which 14 showed a highly complex male karyotype and 5 were normal (46,XY). The predominant leukemic clone was defined as 47,XY,−1,add(4)(q13),+6,add(6)(q21)x2,add(7)(p13),+8,add(8)(q13)x2,−18,−20,+der(?)t(1;?)(q21;?),+mar, ace. This karyotype meets the criteria for a complex monosomal karyotype. Reverse transcriptase PCR screening for 41 common leukemia-associated fusion genes was negative [6]. Targeted next-generation sequencing covering 58 leukemia-associated genes [7] identified a pathogenic *TP53* missense mutation (c.701 A > G, p.Y234C) with a variant allele frequency of 29.7%. The mutation lies within the DNA-binding domain and is known to disrupt p53 function, further indicating an adverse biological profile.

**Fig. 1:**
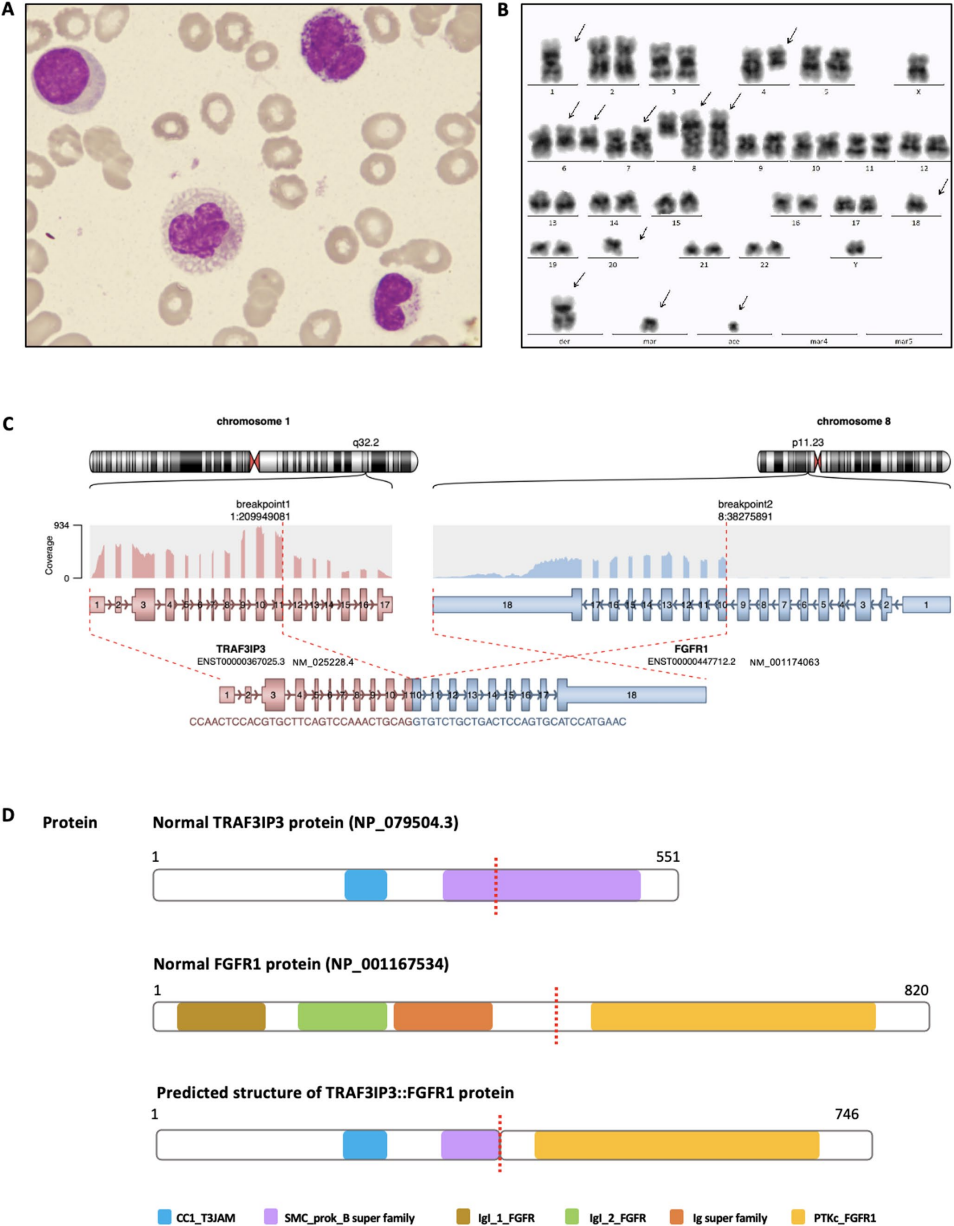
Identification and structure of the *TRAF3IP3*::*FGFR1* fusion. (**A**) Wright-Giemsa-stained bone marrow smear of the patient at initial diagnosis showed monoblasts and promonocytes. (**B**) G-banding revealed a complex monosomal karyotype: 47,XY,−1,add(4)(q13),+6,add(6)(q21)x2,add(7)(p13),+8,add(8)(q13)x2,−18,−20,+der(?)t(1;?)(q21;?),+mar, ace. (**C**) Fusion transcript structure detected by whole transcriptome sequencing, demonstrating an in-frame fusion of exon 11 of *TRAF3IP3* with exon 10 of *FGFR1*. (**D**) Schematic illustration of the wild-type TRAF3IP3 and FGFR1 proteins, and the predicted TRAF3IP3::FGFR1 fusion protein, consisting of 746 amino acids and retaining the CC1_T3JAM domain of TRAF3IP3, as well as the PTKc_FGFR1 domain of FGFR1, indicating preserved dimerization and kinase signaling capacity. Cleavage sites on the 2 proteins are indicated by red dashed line

The patient was diagnosed with AML and initiated on one cycle of HAG chemotherapy (homoharringtonine, cytarabine, and G-CSF), but showed no hematologic response. He subsequently developed severe pulmonary infection. Serial peripheral blood monitoring revealed progressive leukocytosis (WBC 54.22 × 10⁹/L), worsening anemia (Hb 74.8 g/L), and persistent thrombocytopenia (platelets 27.9 × 10⁹/L). Bone marrow reassessment confirmed disease progression, with 92.4% immature monocytic cells. Chest CT demonstrated septic consolidation, and PET-CT showed evidence of extramedullary infiltration. The patient’s condition rapidly deteriorated with neurological symptoms including progressive headache, nausea, and vomiting. Head CT revealed a cerebellar hemorrhage compressing the fourth ventricle with leftward brainstem displacement. He ultimately succumbed to tonsillar herniation only 40 days after initial diagnosis. 

Prior to his death, bone marrow samples had been submitted for whole transcriptome sequencing (WTS), which subsequently revealed a novel in-frame *TRAF3IP3* (exon 11)::*FGFR1* (exon 10) fusion (Fig. 1C). The fusion was further confirmed by RT-PCR using fusion-specific primers, and the breakpoint was validated by Sanger sequencing. The predicted fusion protein consists of 746 amino acids and retains the entire tyrosine kinase domain of FGFR1, suggesting preserved oncogenic signaling capacity (Fig. 1D).

Based on the identification of a pathogenic *FGFR1* fusion, this case is best classified as MLN-TK presenting as *de novo* AML, in line with the latest International Consensus Classification (ICC) and the 5th edition of the World Health Organization Classification of Haematolymphoid Tumours (WHO-HAEM5), which prioritize MLN-TK as a distinct category superseding other myeloid or lymphoid diagnoses in the presence of a relevant kinase rearrangement [1, 2]. However, the concurrent presence of a pathogenic *TP53* mutation and a complex monosomal karyotype introduces diagnostic ambiguity. According to ICC, AML with *TP53* mutations is a recognized entity with distinct clinical implications. While current guidelines prioritize tyrosine kinase rearrangements for MLN-TK classification, the co-occurrence of high-risk genetic lesions suggests that such cases may span multiple diagnostic categories. This highlights the need for cautious interpretation and future refinement of classification frameworks to account for overlapping molecular features.

This case adds *TRAF3IP3* to the growing list of *FGFR1* fusion partners, which currently includes 16 genes including *BCR*, *CNTRL*, *CPSF6*, *CUX1*, *FGFR1OP*, *FGFR1OP2*, *LRRFIP1*, *MYO18A*, *RANBP2*, *SATB1*, *SQSTM1*, *TFG*, *TPR*, *TRIM24*, *ZMYM2*, and *ERVK3*−1 (a human endogenous retroviral sequence) [8–10]. While many *FGFR1*-rearranged neoplasms classically exhibit chronic eosinophilic or mixed-lineage features, an increasing number—including this case—present as *de novo* acute leukemias without eosinophilia or antecedent myeloproliferative history. These forms tend to be rapidly progressive, refractory to conventional therapy, and are associated with poor prognoses [3–5].

The patient harbored a pathogenic *TP53* mutation and a complex monosomal karyotype, both of which are well-established high-risk features in AML and likely served as the primary drivers of chemoresistance and rapid disease progression. *TP53* alterations are enriched in complex karyotype AML and confer marked resistance to standard chemotherapy, often resulting in early relapse or primary induction failure. Similarly, monosomal karyotype—defined by the presence of multiple autosomal losses—has been independently associated with dismal outcomes [11, 12]. The coexisting *TRAF3IP3*::*FGFR1* fusion may have further aggravated the disease course, although its precise contribution remains to be determined.

*TRAF3IP3* (TRAF3 interacting protein 3), located at 1q32.2, encodes a signaling adaptor involved in TNF receptor and NF-κB pathways and has been implicated in early B-cell lymphopoiesis. A recent report described a germline *TRAF3IP3* structural variant in a familial case of B-lymphoblastic leukemia, suggesting a potential role in leukemogenesis [13]. Although this is the first report of *TRAF3IP3* as a fusion partner of *FGFR1*, its N-terminal region contains a predicted coiled-coil domain—a structural feature shared by many transforming *FGFR1* fusion partners—which may facilitate constitutive dimerization and aberrant *FGFR1* kinase activation [4].

Although pemigatinib—a selective *FGFR1*–*3* inhibitor—was not administered in this case due to the rapidly progressive clinical course and delayed sequencing results, its clinical efficacy has been documented in *FGFR1*-rearranged hematologic neoplasms [14]. Given that the *TRAF3IP3*::*FGFR1* fusion retains the intact tyrosine kinase domain, future patients harboring similar fusions may benefit from early molecular diagnosis and timely initiation of *FGFR1*-targeted therapy. In cases achieving complete remission, consolidation with allogeneic hematopoietic stem cell transplantation should be strongly considered, given the poor prognosis associated with *FGFR1*-rearranged leukemias.

Although we confirmed the *TRAF3IP3*::*FGFR1* fusion transcript at base-pair resolution by Sanger sequencing, DNA-based confirmation (e.g., by FISH, optical genome mapping, or whole-genome sequencing) could not be performed due to limited available material. Therefore, while the fusion likely reflects a chromosomal rearrangement, we cannot exclude the possibility of a trans-splicing event. Future studies should investigate this further in similar cases.

To our knowledge, this represents the first documentation of *TRAF3IP3* as a fusion partner of *FGFR1* in hematologic malignancies. This finding further expands the genetic spectrum of *FGFR1*-rearranged neoplasms and underscores the aggressive clinical behavior often associated with this entity.

In conclusion, this case highlights the diagnostic and therapeutic importance of early genomic profiling in aggressive leukemia and raises awareness of potential therapeutic opportunities for patients harboring *FGFR1*-driven fusions.

## Data Availability

No datasets were generated or analysed during the current study.
